# Outcome‐specific risk factors challenge universal dementia prevention priorities: Findings from the Egyptian Dementia Network Registry

**DOI:** 10.1002/alz.71527

**Published:** 2026-06-03

**Authors:** Shimaa A. Heikal, Eman M. Khedr, Gharib Fawi, Mai Othman, Nesma G. Elsheikh, Heba M. Tawfik, Hany I. Hassanin, Sara ElFarrash, Samer Salama, Eman M. Ali, Amna Ibrahim, Ahmed Qayaty, Sara A. Moustafa, Mohamed Salama

**Affiliations:** ^1^ Institute of Global Health and Human Ecology The American University in Cairo Cairo Egypt; ^2^ Neuropsychiatry Department Assiut University Assiut Egypt; ^3^ Neurology Department, Faculty of Medicine Sohag University Sohag Egypt; ^4^ Geriatric Medicine Department, Faculty of Medicine Ain Shams University Cairo Egypt; ^5^ Medical Physiology Department, Faculty of Medicine Mansoura University, and Medical Experimental Research Centre (MERC) Mansoura Egypt; ^6^ Neurology Department, Faculty of Medicine Mansoura University Mansoura Egypt; ^7^ Community Nursing Department, Faculty of Nursing Beni‐Suef University Beni‐Suef Egypt; ^8^ Neurology Department, Faculty of Medicine Beni‐Suef University Beni‐Suef Egypt; ^9^ Neurology Department Beni‐Suef Health Insurance Hospital Beni‐Suef Egypt; ^10^ Global Brain Health Institute (GBHI) Trinity College Dublin Dublin Ireland; ^11^ Clinical Toxicology Department, Faculty of Medicine Mansoura University Mansoura Egypt

**Keywords:** cognitive performance, cognitive reserve, dementia, Egypt, low‐ and middle‐income countries (LMICs), risk factors, social determinants

## Abstract

**BACKGROUND:**

Dementia prevention targets modifiable factors from high‐income cohorts, but most studies conflate dementia status and cognitive performance, distinct outcomes with potentially different outcomes. This distinction is critical to disentangle in resource‐constrained low‐ and middle‐income countries, where resource prioritization requires context‐specific evidence.

**METHODS:**

Cross‐sectional analysis of 660 adults with dementia/mild cognitive impairment from the Egyptian Dementia Network registry (2022 to 2025). Parallel logistic/linear regression models identified factors associated with dementia status versus continuous Mini‐Mental State Examination (MMSE) scores, comparing determinants across outcomes.

**RESULTS:**

The mean age of participants was 68 years, 51% were male, and 81% had a primary education. Multivariable models showed only college education (odds ratio [OR] 0.30, 95% confidence interval [CI]: 0.10 to 0.86) and social inactivity (OR 3.00, 95% CI: 1.27 to 7.08) associated with dementia status. Eight factors (age, diabetes, hypertension, cholesterol, family history, apolipoprotein ε4, body mass index, and sex; all *p* < 0.05) strongly predicted MMSE but not diagnosis.

**DISCUSSION:**

Dementia diagnosis and cognitive performance show mechanistically distinct cross‐sectional associations. LMICs should prioritize education/social engagement for dementia prevention, framing vascular‐metabolic management as cognitive maintenance.

## Highlights

- First LMIC study comparing dementia diagnosis versus cognitive performance.
- Only education/social engagement predicts dementia status independently.
- Vascular/genetic factors strongly predict MMSE but not clinical diagnosis.
- The study challenges universal dementia prevention; reframes LMIC priorities.
- Education and social engagement are high‐yield targets for Egypt/LMICs.

## Graphical abstract

Graphical Abstract illustrating how the different risk factors relate to dementia diagnosis and cognitive performance. Solid arrows indicate robust associations in this cohort; dashed arrows indicate weaker or imprecise associations.

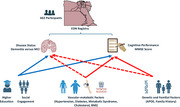

## 1 INTRODUCTION

Dementia is a major global health challenge affecting an estimated 55 million people, with 10 million new cases annually.^1^ Over 60% of dementia cases occur in low‐ and middle‐income countries (LMICs), yet these regions experience profound research gaps.^2^, ^3^ The Lancet Commission identified 14 potentially modifiable risk factors responsible for approximately 45% of dementia cases globally.^4^, ^5^ However, this evidence base derives predominantly from high‐income country cohorts, raising concerns about whether these factors operate similarly across diverse LMIC populations with different social structures, healthcare systems, and exposure patterns.^5^ Recent analyses highlight critical limitations as the commission's 14 risk factors were based on predominantly high‐income country data, and researchers propose an expanded model incorporating four additional LMIC‐relevant factors (poverty, wealth shocks, income inequality, and HIV), which could increase preventable cases to approximately 65% globally.^6^ However, this requires context‐specific evidence from LMIC populations.^6^

LMICs bear a disproportionate dementia burden, yet they have markedly different demographic structures, healthcare access, and educational systems compared to high‐income countries.^7^, ^8^ Egypt exemplifies this challenge, with rapid population aging as people aged more than 65 growing at 2.6% annually, and rising cardiovascular disease burden, yet limited dementia diagnostic capacity and prevention infrastructure.^9^, ^10^ Cost‐constrained health systems require evidence‐based identification of the highest‐impact context‐appropriate risk factors that are feasible to modify at scale.^11^, ^12^ The social determinants of dementia may operate differently in LMICs compared to well‐studied Western populations, where factors such as education, social engagement, occupational patterns, and cardiovascular health may show distinct prevalence, distribution, and strength of association with cognitive outcomes compared to well‐studied Western populations.^13^, ^14^ Additionally, in LMIC settings, health‐system factors, including selective survival to diagnostic age, treatment access, and healthcare recognition, may differentially influence whether pathology translates to clinical diagnosis versus objective test performance.^15^, ^16^ Individuals with severe early‐onset vascular disease may die before reaching diagnostic ages or may remain undiagnosed due to limited specialist services.^15^, ^16^ Similarly, treated hypertension or diabetes in clinic populations may show less apparent association with dementia than in prospective cohort studies, due to survival bias and healthcare‐system selection.^17^

Most dementia epidemiology conflates two distinct outcomes: (1) dementia status, a clinical threshold combining neuropathology, cognitive reserve, and healthcare recognition; and (2) cognitive performance, moment‐to‐moment thinking ability measured continuously.^18^ These outcomes are mechanistically distinct and may have non‐identical determinants.^19^ An individual with substantial pathology but intact cognitive reserve may not meet dementia diagnostic criteria yet still show a measurable decline in objective cognitive testing.^20^, ^21^ Conversely, someone with greater cognitive reserve might maintain high test scores despite harboring pathology.^21^, ^22^ This distinction is clinically important as interventions preventing dementia diagnosis may differ from those optimizing cognitive performance and maintaining independence.^4^

Few dementia studies explicitly compare determinants of disease status versus cognitive performance within the same cohort.^23^, ^24^ This is clinically important because prevention aimed at reducing dementia disease burden may require different interventions than those optimizing cognitive performance in asymptomatic populations.^25^ Evidence from vascular and metabolic research illustrates this divergence as midlife hypertension and type 2 diabetes increase long‐term dementia risk, yet their impact on cross‐sectional cognitive performance depends on blood pressure control, diabetes duration, glycemic variability, and coexisting comorbidities.^26^, ^27^, ^28^ LMIC health systems require identifying which modifiable factors primarily drive dementia incidence versus which primarily affect cognitive performance, so that limited resources can be directed toward the highest‐yield prevention targets.^29^

**RESEARCH IN CONTEXT**

1. **Systematic review**: We conducted a systematic literature review using PubMed, Embase, Scopus, and Google Scholar with terms “dementia risk factors,” “cognitive performance,” “MMSE,” “LMIC,” “Egypt,” “Middle East,” “social determinants,” “vascular risk factors,” and “cognitive reserve.” Most studies derive from high‐income country cohorts and conflate dementia diagnosis with continuous cognitive performance. LMIC data, particularly from Egypt, are limited to small clinic‐based samples lacking standardized outcome comparisons, multicenter designs, or systematic risk factor profiling across diagnostic thresholds versus test performance.
2. **Interpretation**: Our multicenter Egyptian registry analysis (*n* = 660) demonstrates dementia diagnosis and MMSE performance as mechanistically distinct endpoints with divergent risk factor profiles. Only education and social engagement independently predicted dementia status; vascular‐metabolic and genetic factors strongly associated with cognitive performance but not clinical diagnosis. This challenges universal prevention paradigms, identifies context‐specific priorities (education/social engagement), and reframes vascular interventions as cognitive maintenance strategies for resource‐constrained LMIC health systems.
3. **Future directions**: Longitudinal EDN follow‐up should examine MCI‐to‐dementia conversion rates, validate culture‐fair cognitive assessments, and test education/social engagement interventions. Comparative multicenter LMIC studies and mechanistic research on cognitive reserve‐vascular interactions in non‐European populations are needed to develop regionally appropriate dementia prevention strategies for the Middle East and North Africa.

This study compared determinants of dementia diagnosis and continuous Mini‐Mental State Examination (MMSE) scores within the same Egyptian cohort to test whether risk factors operated similarly across disease and performance endpoints. We analyzed data from the Egyptian Dementia Network (EDN) registry to address three objectives: (1) validate the Lancet Commission priorities in an Egyptian population, (2) characterize outcome‐specific associations by comparing whether factors predicting dementia status similarly predict continuous cognitive performance as measured by MMSE scores, and (3) test for differential mechanisms by examining whether divergent associations suggest distinct etiologic pathways to disease versus cognitive decline. A graphical abstract is provided to summarize the conceptual framework and highlight the outcome‐specific associations observed in this study.

## 2 METHODS

### 2.1 Study design and population

This cross‐sectional analysis includes 660 adults with dementia or mild cognitive impairment (MCI) recruited from the EDN registry (2022 to 2025). The EDN is a multicenter observational registry designed to characterize dementia epidemiology, clinical presentation, and risk factors across six Egyptian regions. Participants were aged ≥18 years with dementia or MCI diagnosed according to Diagnostic and Statistical Manual of Mental Disorders‐5th Edition (DSM‐5) or National Institute on Aging‐Alzheimer's Association criteria, confirmed by a consultant neuropsychiatrist. All participants underwent standardized cognitive assessments (MMSE and Montreal Cognitive Assessment [MoCA]), clinical evaluations, and documentation of medical history, smoking, physical activity, social engagement, and family history of dementia.^30^ As a clinic‐based registry, EDN primarily captures symptomatic individuals presenting for evaluation. However, inclusion of participants across the full MMSE spectrum ensures representation of a wide range of cognitive states, from MCI to advanced dementia.

### 2.2 Disease classification and diagnostic criteria

Participants were classified as dementia or MCI. Dementia diagnosis followed DSM‐5 criteria using a standardized protocol incorporating MMSE, functional interviews, clinical assessment, and neuropsychiatrist confirmation. MCI was operationalized using Petersen's criteria (cognitive complaint, objective impairment, preserved general cognition and function, failure to meet dementia criteria).

### 2.3 Primary outcomes

#### 2.3.1 Dementia status (binary): dementia versus MCI

Cognitive performance: MMSE; range 0 to 30). For 83 participants with MoCA scores instead of MMSE, we converted MoCA to MMSE‐equivalent scores using a validated methodology.^31^ Continuous MMSE score captured full cognitive variation independent of diagnostic thresholds.

### 2.4 Predictor variables

**We assessed Lancet Commission‐identified modifiable factors**: education (illiterate/grade school, high school, and college+), social engagement (regular vs minimal social contact), smoking (never, ex‐smoker, current), physical activity (regular vs none), hypertension (self‐reported diagnosis or antihypertensive medication; note: blood pressure not measured), type 2 diabetes (documented diagnosis and laboratory evidence), and high cholesterol (>200 mg/dL or lipid‐lowering medication).

**Additional factors**: Apolipoprotein ε4 (APOE ε4) carrier status (≥1 ε4 allele), family history of dementia (first‐degree relative with dementia diagnosis), dementia medication use (cholinesterase inhibitors or memantine), metabolic syndrome (≥2 conditions), BMI (< 25 vs ≥25 kg/m^2^), and insecticide exposure (self‐reported occupational or household exposure, ever vs never). Dementia medications were not conceptualized as risk factors but were examined descriptively and excluded from multivariable models due to confounding by indication. Demographic covariates included age (continuous), sex, employment status, and marital status.

### 2.5 Statistical analysis

We used multiple imputation by chained equations (MICE) to generate 20 imputed datasets.^32^ For disease‐outcome analyses, all 660 participants with recorded diagnosis were retained; for MMSE analyses, we restricted the analysis to 535 participants with observed MMSE or converted MoCA score. Outcome was not imputed. An adaptive imputation strategy applied predictive mean matching for continuous variables and logistic/multinomial regression for categorical variables.

#### 2.5.1 Sensitivity analyses

To assess the robustness of the findings, we (1) compared results from multiply imputed datasets to complete‐case analysis (excluding participants with any missing data) to examine whether imputation assumptions affected conclusions and (2) examined whether results were sensitive to the choice of reference categories for categorical variables (e.g., “never smoker” vs “smoker”) and to continuous‐to‐categorical transformations for age and other continuous predictors. These sensitivity analyses were performed separately for both dementia status and MMSE models to ensure outcome‐specific patterns were not artifacts of analytic decisions.

#### 2.5.2 Regression models

For each predictor, we conducted separate age/sex‐adjusted logistic regression models predicting dementia status and linear regression models predicting continuous MMSE score, with each model including age and sex as primary covariates and the predictor of interest. Individual models allow systematic examination of associations adjusted for basic demographics before further adjustment for potential confounders in the multivariable stage, providing a comprehensive view of univariable associations prior to addressing collinearity and confounding through multivariable adjustment.

We explicitly compared the direction, magnitude, and statistical significance of associations across the two models (dementia logistic vs MMSE linear) to identify factors showing (1) consistent associations (same direction and similar magnitude across outcomes), (2) divergent associations (different direction or substantially different magnitude), or (3) outcome‐specific associations (statistically significant for one outcome but not the other, with nonoverlapping confidence intervals). This systematic comparison tested the central hypothesis that dementia status and continuous cognitive performance shared distinct etiologic pathways and might be influenced by different sets of modifiable and non‐modifiable factors.

Results from multiply imputed data were pooled using Rubin's rules. We report odds ratios (ORs) with 95% confidence intervals (CIs) and *p* values for logistic regression and unstandardized beta coefficients (in MMSE points) with 95% CI and *p* values for linear regression.

All analyses used R version 4.5.2 (packages: mice, broom, dplyr, forcats, ggplot2). Code is available from the corresponding author upon request.

## 3 RESULTS

### 3.1 Study population

Of 662 registry participants, 660 met criteria for dementia or MCI and were included in the disease‑outcome analyses; two were excluded due to missing diagnosis. Among included participants, 609 (92.3%) had dementia, and 51 (7.7%) had MCI/normal cognition. Despite the relatively small proportion of MCI cases, MMSE scores spanned the full cognitive range, capturing individuals with mild, moderate, and advanced impairment. This continuous distribution supports the use of MMSE as a dimensional measure reflecting the broader cognitive spectrum within the cohort. Mean age was 68.4 years (SD 10.1). Slightly more than half were male (51%). Most had low educational attainment: 370 (81%) had illiteracy/grade‑school education, 45 (9.9%) high school, and 40 (8.8%) college or higher. Social engagement data were available for 363 participants, of whom 179 (49.3%) reported regular social activity (Table 1). Data missingness ranged from 0% to 87.6%. Because most models focused on education, social activity, and APOE **ε**4, we emphasize missingness for these variables here: 31.1% for education, 45.0% for social activity, and 87.6% for APOE **ε**4. MICE generated 20 complete datasets for analysis.

**TABLE 1:** Demographic and clinical characteristics of study population.

Characteristic	Diseaseoutcome sample (*n* = 660)	MMSE analysis sample (*n* = 535)
**Age, years**		
Mean (SD)	68.4 (10.1)	68.3 (10.2)
**Sex**		
Female, *n* (%)	324 (49.0)	264 (49.3)
Male, *n* (%)	335 (51.0)	271 (50.7)
**Education**		
Illiterate/grade school, *n* (%)	370 (81.0)	368 (81.4)
High school, *n* (%)	45 (9.9)	44 (9.7)
College or higher, *n* (%)	40 (8.8)	40 (8.8)
**Smoking status**		
Nonsmoker, *n* (%)	458 (85.0)	353 (82.9)
Current smoker, *n* (%)	47 (11.0)	47 (11.0)
Exsmoker, *n* (%)	26 (6.1)	26 (6.1)
**Physical inactivity**		
Active, *n* (%)	90 (24.9)	90 (24.9)
Inactive, *n* (%)	272 (75.0)	271 (75.1)
**BMI**		
Lower BMI (underweight/normal), *n* (%)	170 (44.0)	167 (43.8)
Higher BMI (overweight/obese), *n* (%)	217 (56.0)	214 (56.2)
**Hypertension**		
No, *n* (%)	296 (44.9)	218 (40.7)
Yes, *n* (%)	364 (55.2)	317 (59.3)
**Diabetes**		
No, *n* (%)	505 (76.6)	408 (76.3)
Yes, *n* (%)	155 (23.5)	127 (23.7)
**High cholesterol**		
No, *n* (%)	555 (84.1)	450 (84.1)
Yes, *n* (%)	105 (15.9)	85 (15.9)
**Metabolic syndrome (≥2 conditions)**		
No, *n* (%)	429 (65.0)	391 (73.1)
Yes, *n* (%)	231 (35.0)	144 (26.9)
**Cerebrovascular disease**		
No, *n* (%)	512 (95.7)	512 (95.7)
Yes, *n* (%)	23 (4.3)	23 (4.3)
**APOE ε4 carrier**		
Noncarrier, *n* (%)	67 (84.7)	53 (84.1)
Carrier, *n* (%)	15 (18.3)	10 (15.9)
**Insecticide exposure**		
No, *n* (%)	494 (74.9)	370 (69.2)
Yes, *n* (%)	166 (25.2)	165 (30.8)
**Social activity**		
Yes, *n* (%)	179 (49)	178 (49.2)
No, *n* (%)	184 (51.0)	184 (50.8)
**Marital status**		
Married, *n* (%)	250 (72.3)	249 (72.2)
Other (widowed/single/divorced), *n* (%)	96 (27.8)	96 (27.8)
**Employment status**		
Working, *n* (%)	92 (20.4)	90 (20.3)
Not working/retired/housewife, *n* (%)	359 (79.6)	353 (79.7)
**Family history of dementia**		
Negative, *n* (%)	313 (78.6)	306 (82.5)
Positive, *n* (%)	85 (21.4)	65 (17.5)
**Dementia medication use**		
No, *n* (%)	198 (75.6)	197 (75.5)
Yes, *n* (%)	64 (24.5)	64 (24.5)
**MMSE (or converted MoCA)**		
Observed MMSE, *n*	–	535
Mean (SD)	–	16.2 (7.0)

**FIGURE 1:**
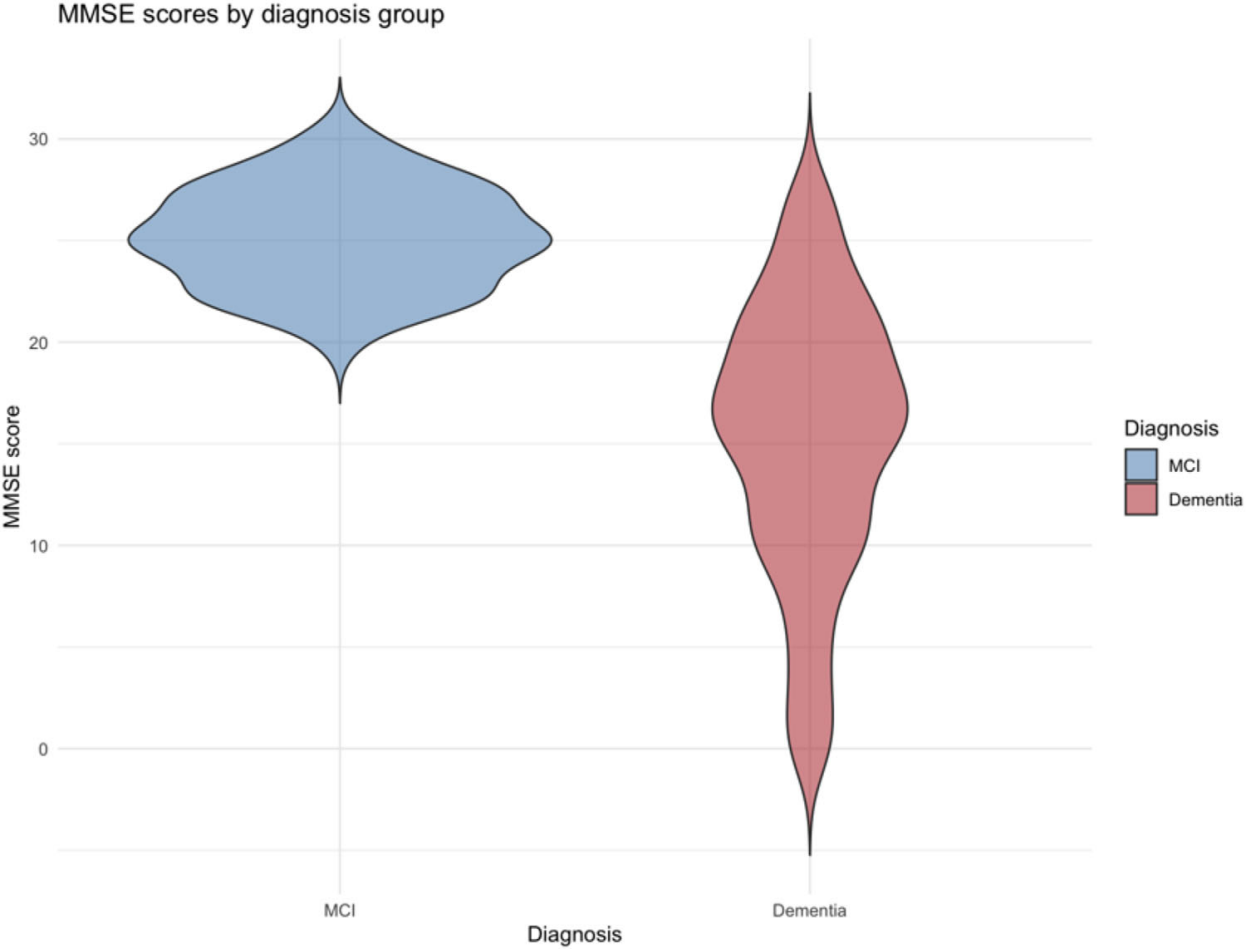
Distribution of MMSE scores by diagnosis (MCI vs dementia).

For the cognitive analyses, we restricted to participants with observed MMSE data. Of the 662 participants, 535 (80.8%) had a recorded MMSE‑equivalent score: 458 had an MMSE score, and 83 had a MoCA score that was converted to MMSE units; participants with both tests contributed a single value. The remaining 127 (19.2%) without MMSE data were excluded from MMSE models. Among the 535 participants with MMSE data, scores were substantially lower in the dementia group than in the MCI group, with notable overlap in distributions (Figure 1). Mean MMSE was 16.2 (SD 7.0), and mean age was 68.3 years (SD 10.2); 271 (50.7%) were male, and 368 (81.4%), 44 (9.7%), and 40 (8.8%) had illiteracy/grade school, high school, and college‑level education, respectively. Social‑activity information was available for 362 participants, of whom 178 (49.2%) reported regular social engagement (Table 1). For key MMSE‑model predictors, missingness was 15.5% for education, 32.3% for social activity, and 88.2% for APOE **ε**4. Multiple imputation (20 datasets) was applied to these predictors but not to the MMSE outcome.

### 3.2 Individual regression analysis

**FIGURE 2:**
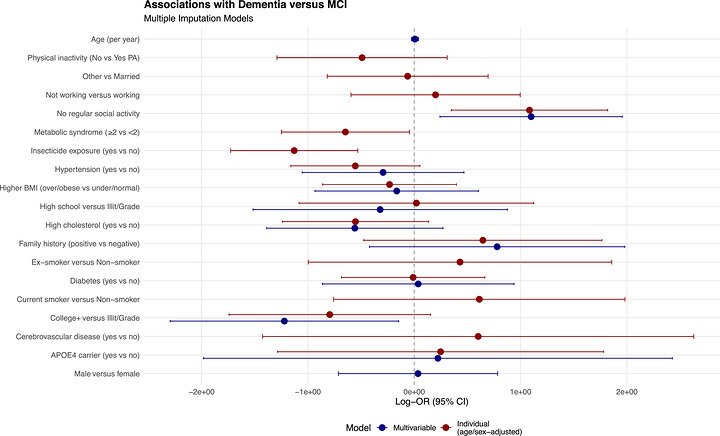
Forest plot of individual logistic regression models for dementia status (dementia vs MCI). Odds ratios (ORs) with 95% confidence intervals are presented on a log scale; the vertical line indicates no association (OR = 1).

In age/sex‐adjusted logistic regression models, college‐level education was associated with substantially lower odds of dementia (OR 0.40, 95% CI: 0.18 to 0.89), as were metabolic syndrome (OR 0.52, 95% CI: 0.29 to 0.95) and insecticide exposure (OR 0.32, 95% CI: 0.18 to 0.59) (Table 2; Figure 2). Social inactivity was strongly associated with increased dementia odds (OR 2.95, 95% CI: 1.51 to 5.74). Other associations (smoking, hypertension, diabetes, cholesterol, and APOE **ε**4) were directionally consistent with prior literature but imprecise, with confidence intervals crossing unity.

**TABLE 2:** Individual regression models for dementia (OR) and MMSE (β).

Predictor	Dementia OR (95% CI)	MMSE β (95% CI, points)
High school education	0.99 (0.33 to 2.98)	+1.09 (0.64 to 1.55)
College+ education	0.40 (0.18 to 0.89)	+1.72 (1.26 to 2.18)
Current smoking	3.24 (0.73 to 14.43)	−2.34 (−2.74 to −1.93)
Previous smoking	1.67 (0.37 to 7.53)	−4.21 (−4.69 to −3.72)
Physical inactivity	0.58 (0.25 to 1.33)	+0.69 (0.38 to 1.00)
Higher body mass index (≥ 25)	0.76 (0.40 to 1.43)	−0.10 (−0.36 to 0.17)
Hypertension	0.57 (0.31 to 1.05)	+2.05 (1.79 to 2.32)
Diabetes	0.99 (0.50 to 1.94)	−1.84 (−2.15 to −1.53)
High cholesterol	0.57 (0.29 to 1.14)	+0.38 (0.02 to 0.74)
Metabolic syndrome (>2 comorbidities)	0.52 (0.29 to 0.95)	−0.76 (−1.06 to −0.46)
Cerebrovascular disease	1.82 (0.24 to 13.84)	−2.12 (−2.78 to −1.47)
APOE ε4 carrier	1.26 (0.30 to 5.40)	−1.10 (−1.42 to −0.78)
Insecticide exposure	0.32 (0.18 to 0.59)	+5.48 (5.20 to 5.76)
Social inactivity	2.95 (1.51 to 5.74)	−4.52 (−4.77 to −4.26)
Marital status (not married)	0.94 (0.46 to 1.91)	+0.13 (−0.19 to 0.44)
Employment status (not working)	1.25 (0.55 to 2.84)	+0.25 (−0.11 to 0.60)
Positive family history	1.85 (0.66 to 5.17)	+1.40 (1.06 to 1.73)
Dementia medications	–	−4.36 (−4.63 to −4.09)

**FIGURE 3:**
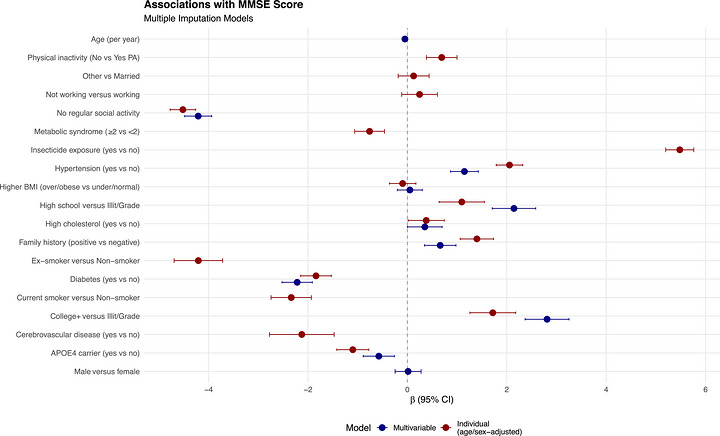
Forest plot of multivariable linear regression models for MMSE score. Beta coefficients (*β*) represent change in MMSE score associated with each predictor; vertical line indicates no association (*β* = 0).

In linear regression models for MMSE, higher education was associated with better cognition: high school (*β*: 1.09, 95% CI: 0.64 to 1.55) and college level (*β*: 1.72, 95% CI: 1.26 to 2.18) (Table 2; Figure 3). In contrast, current and ex‐smoking (*β*: −2.34 and −4.21, respectively), diabetes (*β*: −1.84, 95% CI: −2.15 to −1.53), metabolic syndrome (*β*: −0.76, 95% CI: −1.06 to −0.46), cerebrovascular disease (*β*: −2.12, 95% CI: −2.78 to −1.47), APOE **ε**4 carriership (*β*: −1.10, 95% CI: −1.42 to −0.78), social inactivity (*β*: −4.52, 95% CI: −4.77 to −4.26) were associated with lower MMSE scores. Dementia medication use (*β*: −4.36, 95% CI: −4.63 to −4.09) was also associated with lower MMSE scores, which likely reflects confounding by indication, as medications are prescribed to individuals with more severe impairment. Hypertension (*β*: 2.05, 95% CI: 1.79 to 2.32), high cholesterol (*β*: 0.38, 95% CI: 0.02 to 0.74), and positive family history (*β*: 1.40, 95% CI: 1.06 to 1.73) showed modest positive associations with MMSE.

### 3.3 Multivariable regression analysis

*Dementia status*: In fully adjusted models, only two factors remained clearly associated with dementia: college‐level education (OR 0.30, 95% CI: 0.10 to 0.86) and social inactivity (OR 3.00, 95% CI: 1.27 to 7.08) (Table 3; Figure 2). Other covariates showed directions consistent with prior literature, but 95% CIs crossed unity.

**TABLE 3:** Comparison of multivariable associations with dementia status and MMSE.

Predictor	Dementia OR	Dementia 95% CI	Dementia *p* value	MMSE β (points)	MMSE 95% CI	MMSE *p* value
Age	1.01	0.97 to 1.04	0.68	−0.05	−0.06 to −0.04	9.96 × 10^−^^1^ ^5^
Male versus female	1.04	0.49 to 2.19	0.93	+0.01	−0.25 to 0.28	0.91
High school	0.73	0.22 to 2.40	0.60	+2.14	1.71 to 2.58	8.03 × 10^−^^2^ ^2^
College+	0.30	0.10 to 0.86	0.026	+2.81	2.37 to 3.25	1.65 × 10^−^^3^ ^5^
Eversmoker	1.62	0.49 to 5.36	0.42	–	–	–
Social inactivity	3.00	1.27 to 7.08	0.012	−4.21	−4.48 to −3.94	1.60 × 10^−^^1^ ^9^ ^4^
Diabetes	1.04	0.42 to 2.56	0.93	−2.22	−2.52 to −1.91	1.06 × 10^−^^4^ ^5^
Higher BMI	0.85	0.39 to 1.83	0.67	+0.05	−0.20 to 0.30	0.70
Hypertension	0.75	0.35 to 1.60	0.45	+1.15	0.87 to 1.43	9.58 × 10^−^^1^ ^6^
High cholesterol	0.57	0.25 to 1.31	0.19	+0.35	0.00 to 0.70	0.049
Positive family history	2.18	0.66 to 7.24	0.20	+0.66	0.34 to 0.98	4.37 × 10^−^^5^
APOE ε4 carrier	1.25	0.14 to 11.33	0.84	−0.57	−0.89 to −0.26	3.42 × 10^−^^4^

*MMSE score*: In multivariable models, both high school (*β*: 2.14, 95% CI: 1.71 to 2.58) and college‐level education (*β*: 2.81, 95% CI: 2.37 to 3.25) were associated with higher MMSE scores, consistent with a graded cognitive‐reserve effect (Table 3; Figure 3). Social inactivity remained strongly associated with poorer cognition (*β*: −4.21, 95% CI: −4.48 to −3.94). Diabetes (*β*: −2.22, 95% CI: −2.52 to −1.91) and APOE **ε**4 carriership (*β*: −0.57, 95% CI: −0.89 to −0.26) were associated with lower MMSE. In contrast, hypertension (*β*: 1.15, 95% CI: 0.87 to 1.43), high cholesterol (*β*: 0.35, 95% CI: 0.00 to 0.70), and positive family history (*β*: 0.66, 95% CI: 0.34 to 0.98) showed modest positive associations. Age showed a small, consistent negative association (*β*: −0.05 per year, 95% CI: −0.06 to −0.04). Finally, given its implausible direction and likely bias in individual analysis, insecticide exposure was excluded from multivariable models.

### 3.4 Summary of outcome‐specific patterns

Risk factor profiles diverged substantially across outcomes (Table 3). In multivariable models predicting dementia status, only two predictors achieved statistical significance (*p* < 0.05): college‐level education (OR 0.30, 95% CI: 0.10 to 0.86) and social inactivity (OR 3.00, 95% CI: 1.27 to 7.08). All other factors had confidence intervals crossing unity and *p* values > 0.18.

In contrast, the same model predicting continuous MMSE scores showed eight factors with highly significant associations (*p* < 0.05, most *p* < 0.001): age, both educational levels, social inactivity, diabetes, hypertension, high cholesterol, family history, and APOE **ε**4 carriership (Table 3). The marked divergence between dementia‐status associations and MMSE associations suggests that factors strongly predicting objective test‐based cognition in older adults may not independently predict who crosses diagnostic thresholds for ADRD, with critical implications for prevention strategies in LMIC.

## 4 DISCUSSION

This study compared determinants of dementia status and continuous cognitive performance in a LMIC setting. Education and social inactivity were shared determinants, consistently associated with both dementia status and MMSE performance.^4^, ^22^, ^33^ In contrast, vascular–metabolic and genetic factors (diabetes, hypertension, high cholesterol, family history, and APOE **ε**4) showed outcome‐specific associations, relating strongly to MMSE but not independently to dementia status. These findings should be interpreted as cross‐sectional associations rather than evidence of causal risk or protective effects.

The divergence between dementia status and MMSE performance reflects fundamental differences in what each outcome captures. Dementia status is a composite endpoint combining neuropathology, cognitive reserve, functional decline, healthcare recognition, and documented diagnosis.^34^ In contrast, MMSE scores reflect moment‐to‐moment cognitive test performance, sensitive to pathological burden, current metabolic and vascular function, mood, medication effects, education, and cognitive stimulation.^19^, ^35^

In LMIC clinic‐based populations, several factors amplify outcome‐specific divergence. First, cross‐sectional dementia cases represent survivors to diagnostic age; individuals with severe early‐onset vascular disease may have died before reaching diagnostic ages, artificially attenuating vascular risk associations with disease status.^15^ Second, treated vascular conditions may paradoxically show positive associations with cognition due to healthcare‐system selection: Individuals with well‐controlled hypertension represent a self‐selected, health‐conscious group with better medication adherence and health behaviors.^17^ Third, education's influence on test performance is pronounced in low‐literacy populations, where educational attainment may strongly predict MMSE scores even when controlling for underlying cognitive pathology.^21^

Social inactivity nearly tripled the odds of dementia and was associated with one of the largest decrements in MMSE, comparable to the benefit of higher educational attainment. This supports evidence highlighting social isolation as a major modifiable dementia risk factor and extends it to a Middle Eastern LMIC context, where traditional family structures are changing with urbanization.^4^, ^36^ The strong, consistent association across outcomes suggests social engagement influences both disease mechanisms (neuroinflammation, stress, and depression) and real‐time cognitive performance through enrichment and stimulation.^22^, ^37^, ^38^

In contrast, vascular, metabolic, and genetic factors showed striking divergence between dementia status and MMSE performance. Cross‐sectional dementia diagnosis reflects not only exposure history but also survival and clinical recognition, potentially underestimating vascular risk contributions in settings with high premature mortality and limited diagnostic capacity.^8^, ^17^ Individuals with severe vascular disease may die before reaching diagnostic ages or remain undiagnosed due to limited specialist services.^7^, ^39^ Additionally, antihypertensive and cardiometabolic treatment could attenuate vascular dementia relationships while still influencing cognition via blood pressure variability, hypoperfusion, or medication side effects.^27^, ^28^ The negative effects of vascular and metabolic dysregulation are evident especially in LMIC populations with high burdens of untreated cardiovascular disease.^40^

The finding that hypertension and high cholesterol were associated with higher MMSE scores, despite null associations with dementia, echoes prior work reporting “paradoxical” protective associations of late‐life vascular factors with cognition, often attributed to survivor bias and treatment effects.^26^, ^41^ These patterns may be amplified in our clinic‐based cohort, where participants typically have greater healthcare access than the general older Egyptian population, potentially introducing selection bias.^17^, ^28^ APOE **ε**4 carriership was associated with lower MMSE but not with dementia status, potentially due to limited statistical power from very high missingness and ethnic differences in APOE–dementia relationships in non‐European populations.^13^

In individual models, insecticide exposure appeared strongly protective for both dementia and MMSE, suggesting better cognition among exposed individuals; this is biologically implausible and conflicts with toxicological evidence.^42^, ^43^ We therefore treated this finding as artifactual and excluded insecticide exposure from multivariable models to avoid distorting other estimates. Several mechanistic explanations account for this paradoxical signal. First, reverse causality: Cognitively impaired individuals or their caregivers may reduce insecticide use due to forgetfulness or poor recall of exposure history. Second, recall bias: Participants with more severe cognitive impairment or advanced dementia may have difficulty accurately remembering or reporting past insecticide exposure, leading to differential underreporting in the dementia group compared to those with milder MCI.^19^, ^44^ Third, occupational health selection: Participants reporting high insecticide exposure likely include active farmers and agricultural workers with ongoing employment, occupational engagement, and associated protective factors, including income stability, structured daily activity, and cognitive stimulation from work, that may buffer cognitive decline independently of exposure status.^45^ Fourth, survivor bias in agricultural communities, as individuals in farming occupations with chronic pesticide exposure who develop severe cognitive or vascular disease may have already died or dropped out of the clinic‑based sample, leaving a surviving cohort of more cognitively resilient exposed workers.^46^ These explanations remain hypotheses rather than conclusions, and prospective studies incorporating objective biomarker‑based exposure measurement are needed to clarify this signal in Egyptian settings where household and agricultural pesticide use is widespread.

Dementia medication use was associated with markedly lower MMSE, reflecting confounding by indication.^47^ We did not include dementia treatment in multivariable models to avoid obscuring upstream risk factor relationships.

### 4.1 Implications for dementia prevention in LMICs

Our findings have direct implications for health policy in Egypt and similar LMICs. Robust associations of education and social engagement with both dementia and cognitive performance support prioritizing early‐life schooling (particularly female education), adult literacy programs, and community‐based social engagement initiatives as cornerstone prevention strategies.^4^, ^14^ These factors are modifiable and culturally feasible and align with existing family networks.

Our outcome‐specific findings carry particular significance for Alzheimer's Disease and Related Dementias (ADRD) prevention in LMICs, where ADRD represents the predominant dementia form, yet prevention strategies have been overwhelmingly developed in Western, high‐resource populations.^1^, ^2^, ^3^ Our results suggest substantial reorientation of Alzheimer's prevention priorities in resource‐constrained settings.

First, cognitive reserve building. Cognitive reserve, built through education and sustained by social engagement, emerges as a robust protective pathway across diagnostic and functional cognitive domains. This aligns with cognitive reserve theory and supports population‐level cognitive reserve building as foundational for Alzheimer's prevention in Egypt and similar settings.^1^, ^4^ Female education represents a high‐leverage target, given women account for 65% to 70% of Alzheimer's cases globally and have lower LMIC educational attainment.^5^ These investments are cost‐effective, align with social structures, and support the UN Sustainable Development Goals.

Second, reframing vascular‐metabolic interventions. The outcome‐specific vascular‐metabolic pattern suggests reframing from dementia prevention to cognitive maintenance. Rather than marketing hypertension/diabetes control as dementia prevention, health systems should emphasize that vascular management maintains cognitive function, independence, and quality of life.^17^ Weak associations with dementia diagnosis but strong cognitive test associations suggest vascular interventions maintain functional cognition rather than prevent Alzheimer's diagnostic thresholds. This reframing promotes cardiovascular management for healthy cognitive aging, improving adherence where pharmaceutical dementia treatments are limited.^6^

Third, leveraging social engagement. The strong social engagement effect (nearly tripling dementia risk when absent) points to an overlooked intervention pathway: community‐based cognitive stimulation, intergenerational programs, and technology‐enabled connection. These are relevant in rapidly urbanizing Egypt, where traditional family‐based social structures are undergoing a transformation.^48^, ^49^ Social isolation is a major modifiable risk factor amenable to low‐cost community interventions.

Future trials should include both endpoints; cognitive performance measures may show effects more readily than incident dementia and be culturally appropriate with culture‐fair assessments developed.^18^, ^22^

### 4.2 Strengths and limitations

This study is among the first from a multicenter Egyptian dementia registry to jointly examine determinants of dementia status and MMSE within the same cohort. We applied standardized diagnostic criteria and focused on Lancet Commission‐prioritized risk factors.^4^, ^5^

However, several limitations warrant caution. The primary limitation of this study is its cross‐sectional design, which precludes causal inference and is vulnerable to reverse causality and survivor bias, particularly for vascular‐metabolic factors.^17^ The clinic‐based sample may not represent the broader Egyptian older population and likely overrepresents individuals with advanced disease and better access to specialty care. Additionally, although MCI cases were underrepresented (7.7%), reflecting healthcare access patterns and diagnostic pathways in Egypt, the cohort included participants across the full MMSE range. This distribution captures variability from mild impairment to advanced dementia and partially mitigates concerns regarding restricted cognitive representation. However, the MCI subgroup may still not be representative of community‐based MCI populations, particularly milder or undiagnosed cases.

Moreover, missingness was high for some variables (APOE **ε**4, social activity), and although multiple imputation mitigates bias under missing‐at‐random assumptions, residual bias is possible.^32^ MMSE is influenced by education, literacy, and cultural factors, particularly relevant in our low‐literacy sample, underscoring the need for more culture‐fair cognitive assessments in future Egyptian studies.^19^ Finally, the limited sample size reduced power to detect modest effects for some predictors, especially genetic and cerebrovascular variables.

Future work should extend these analyses to longitudinal follow‐up within the EDN registry, allowing examination of incident dementia, cognitive trajectories, and MCI to dementia transitions. Incorporating more granular measures of social networks, depression, life‐course education, and objective vascular control will help clarify causal pathways. Comparative analyses across LMIC cohorts in the Middle East and North Africa could test whether observed patterns generalize across diverse contexts.

## 5 CONCLUSION

In this multicenter Egyptian registry, education and social engagement consistently distinguished both dementia status and cognitive performance, whereas vascular‐metabolic and genetic factors were more strongly linked to MMSE variation than to crossing the clinical threshold for dementia. These outcome‐specific patterns support conceptualizing dementia status and continuous cognition as related but non‐interchangeable endpoints in LMIC research on dementia prevention. Investments in early‐life education, adult literacy, and age‐friendly opportunities for social participation, alongside strengthened detection and management of cardiometabolic conditions, may offer complementary routes to maintaining cognitive health and delaying disability in aging populations in Egypt and similar settings. Recognition of these distinct pathways is essential for developing dementia prevention strategies appropriate to LMIC contexts where diagnostic capacity and pharmaceutical resources are limited. Longitudinal follow‐up within EDN and other LMIC cohorts is needed to confirm these findings, clarify causal pathways, and inform integrated dementia prevention strategies spanning cognitive reserve, social connectedness, and vascular risk control.

## AUTHOR CONTRIBUTIONS

Shimaa A. Heikal designed the study, conducted statistical analyses, and drafted the manuscript. Shimaa A. Heikal, Gharib Fawi, Eman M. Khedr, Mai Othman, Sara A. Moustafa, Sara ElFarrash, Nesma G. Elsheikh, Heba M. Tawfik, Eman M. Ali, Amna Ibrahim, Ahmed Qayaty, and Hany I. Hassanin contributed to data collection. Mohamed Salama critically reviewed and edited the manuscript. All authors read and approved the final version of the manuscript.

## CONFLICT OF INTEREST STATEMENT

The authors declare no conflict of interest. Author disclosures are available in the Supporting Information.

## CONSENT

All participants or legal guardians provided written informed consent following the Declaration of Helsinki. All protocols were approved by the Institutional Review Board at the American University in Cairo (IRB‐AUC# 2024‐2025‐028).

## Supporting information



Supporting Information

## References

[1] WHO . Dementia. 2025; published online March. (accessed Dec 6, 2025). https://www.who.int/news‐room/fact‐sheets/detail/dementia

[2] Heikal SA , Salama M . Epidemiology of dementia of the world's two‐thirds. In: Dementia Care and Provision in the Majority World. 1st ed. Routledge; 2025:11‐22. doi:10.4324/9781003535997

[3] WHO . WHO | Global action plan on the public health response to dementia 2017 ‐ 2025. WHO. 2017.

[4] Livingston G , Huntley J , Sommerlad A , et al. Dementia prevention, intervention, and care: 2020 report of the Lancet Commission. Lancet. 2020;396:413‐446. doi:10.1016/S0140‐6736(20)30367‐6 32738937 10.1016/S0140-6736(20)30367-6 PMC7392084

[5] Livingston G , Huntley J , Liu KY , et al. Dementia prevention, intervention, and care: 2024 report of the Lancet standing Commission. Lancet. 2024;404:572‐628. doi:10.1016/S0140‐6736(24)01296‐0 39096926 10.1016/S0140-6736(24)01296-0

[6] Mostert CM , Udeh‐Momoh C , Winkler AS , et al. Broadening dementia risk models: building on the 2024 Lancet Commission report for a more inclusive global framework. EBioMedicine. 2025;120:105950. doi:10.1016/j.ebiom.2025.105950 41004922 10.1016/j.ebiom.2025.105950 PMC12509747

[7] Ferri CP , Jacob KS . Dementia in low‐income and middle‐income countries: different realities mandate tailored solutions. PLoS Med. 2017;14:e1002271. doi:10.1371/journal.pmed.1002271 28350797 10.1371/journal.pmed.1002271 PMC5370095

[8] Walker R , Paddick S‐M . Dementia prevention in low‐income and middle‐income countries: a cautious step forward. Lancet Glob Health. 2019;7:e538‐9. doi:10.1016/S2214‐109X(19)30169‐X 31000118 10.1016/S2214-109X(19)30169-X

[9] Rakab MS , Baklola M , Elsalakawi BH , et al. Ischemic heart disease awareness in Egypt's aging population: findings from a national cross‐sectional study. Egypt. Heart J. 2024;76:152. doi:10.1186/s43044‐024‐00584‐1 39576408 10.1186/s43044-024-00584-1 PMC11584834

[10] Moustafa SA , Boersch‐Supan A , Salama M . Aging in an Arab country: knowledge gaps in Egypt. Nat Aging. 2023;3:1042‐1044. doi:10.1038/s43587‐023‐00484‐0 37605087 10.1038/s43587-023-00484-0

[11] Kenne Malaha A , Thébaut C , Achille D , Preux P‐M , Guerchet M . Costs of dementia in low‐and middle‐income countries: a systematic review. J Alzheimers Dis. 2023;91:115‐128. doi:10.3233/JAD‐220239 36404540 10.3233/JAD-220239

[12] Rodriguez FS , Roehr S . Challenges in dementia risk prediction in low‐income and middle‐income countries. Lancet Glob Health. 2020;8:e458‐9. doi:10.1016/S2214‐109X(20)30077‐2 32199107 10.1016/S2214-109X(20)30077-2

[13] Mukadam N , Sommerlad A , Huntley J , Livingston G . Population attributable fractions for risk factors for dementia in low‐income and middle‐income countries: an analysis using cross‐sectional survey data. Lancet Glob Health. 2019;7:e596‐603. doi:10.1016/S2214‐109X(19)30074‐9 31000129 10.1016/S2214-109X(19)30074-9 PMC7617123

[14] Stephan BCM , Pakpahan E , Siervo M , et al. Prediction of dementia risk in low‐income and middle‐income countries (the 10/66 Study): an independent external validation of existing models. Lancet Glob Health. 2020;8:e524‐35. doi:10.1016/S2214‐109X(20)30062‐0 32199121 10.1016/S2214-109X(20)30062-0 PMC7090906

[15] Luo H , Koponen M , Roethlein C , et al. A multinational cohort study of trends in survival following dementia diagnosis. Commun Med. 2025;5:203. doi:10.1038/s43856‐025‐00923‐6 40437158 10.1038/s43856-025-00923-6 PMC12120012

[16] Rountree SD , Chan W , Pavlik VN , Darby EJ , Doody RS . Factors that influence survival in a probable Alzheimer disease cohort. Alzheimers Res Ther. 2012;4:16. doi:10.1186/alzrt119 22594761 10.1186/alzrt119 PMC3506931

[17] Mcgrath ER , Beiser AS , O'Donnell A , et al. Determining vascular risk factors for dementia and dementia risk prediction across mid‐ to later life: the Framingham Heart Study. Neurology. 2022;99:E142‐53. doi:10.1212/WNL.0000000000200521 35584926 10.1212/WNL.0000000000200521 PMC9280997

[18] Dubois B , Hampel H , Feldman HH , et al. Preclinical Alzheimer's disease: definition, natural history, and diagnostic criteria. Alzheimers Dement. 2016;12:292‐323. doi:10.1016/j.jalz.2016.02.002 27012484 10.1016/j.jalz.2016.02.002 PMC6417794

[19] Gluhm S , Goldstein J , Loc K , Colt A , Liew CV , Corey‐Bloom J . Cognitive performance on the mini‐mental state examination and the montreal cognitive assessment across the healthy adult lifespan. Cogn Behav Neurol. 2013;26:1‐5. doi:10.1097/WNN.0b013e31828b7d26 23538566 10.1097/WNN.0b013e31828b7d26 PMC3638088

[20] Beveridge J , Sheth P , Thakkar S , Silverglate B , Grossberg G . The impact of cognitive reserve relative to risk of Alzheimer's disease and rate of progression: an up‐to‐date review of the literature. Expert Rev Neurother. 2025;25:175‐187. doi:10.1080/14737175.2024.2445015 39698839 10.1080/14737175.2024.2445015

[21] Marmor A , Meiner Z , Merhavi SK , Vakil E . Cognitive reserve as a predictor of cognitive decline, but not age of diagnosis in patients with possible young‐onset Alzheimer's disease: an underexplored population. J Alzheimers Dis Rep. 2025;9:25424823251383904. 10.1177/25424823251383903 PMC12579165 41180956

[22] Stern Y , Barnes CA , Grady C , Jones RN , Raz N . Brain reserve, cognitive reserve, compensation, and maintenance: operationalization, validity, and mechanisms of cognitive resilience. Neurobiol Aging. 2019;83:124. doi:10.1016/j.neurobiolaging.2019.03.022 31732015 10.1016/j.neurobiolaging.2019.03.022 PMC6859943

[23] Aiello AE , Momkus J , Stebbins RC , et al. Risk factors for Alzheimer's disease and cognitive function before middle age in a U.S. representative population‐based study. Lancet Reg Health Am. 2025;45:101087. doi:10.1016/j.lana.2025.101087 40242320 10.1016/j.lana.2025.101087 PMC12001091

[24] Villalpando JM , Leclerc BS , Le MT , Hudon C , Bolduc A , Kergoat MJ . A comparison of clinical diagnostic classification criteria used in longitudinal cohort studies of the Alzheimer's disease continuum: a systematic review. Neuropsychol Rev. 2025:1‐22. doi:10.1007/s11065‐025‐09663‐9. Published online May 9, 2025. 40343672 10.1007/s11065-025-09663-9 PMC13279743

[25] Harada CN , Natelson Love MC , Triebel KL . Normal cognitive aging. Clin Geriatr Med. 2013;29:737‐752. doi:10.1016/j.cger.2013.07.002 24094294 10.1016/j.cger.2013.07.002 PMC4015335

[26] Iadecola C , Yaffe K , Biller J , et al. Impact of hypertension on cognitive function: a scientific statement from the American Heart Association. Hypertension. 2016;68:e67‐e94. doi:10.1161/HYP.0000000000000053 27977393 10.1161/HYP.0000000000000053 PMC5361411

[27] Koekkoek PS , Kappelle LJ , van den Berg E , Rutten G , Biessels GJ . Cognitive function in patients with diabetes mellitus: guidance for daily care. Lancet Neurol. 2015;14:329‐340. doi:10.1016/S1474‐4422(14)70249‐2 25728442 10.1016/S1474-4422(14)70249-2

[28] McWhirter L , Ritchie C , Stone J , Carson A . Functional cognitive disorders: a systematic review. Lancet Psychiatry. 2020;7:191‐207. doi:10.1016/S2215‐0366(19)30405‐5 31732482 10.1016/S2215-0366(19)30405-5

[29] Sperling RA , Aisen PS , Beckett LA , et al. Toward defining the preclinical stages of Alzheimer's disease: recommendations from the National Institute on Aging‐Alzheimer's Association workgroups on diagnostic guidelines for Alzheimer's disease. Alzheimers Dement. 2011;7:280‐292. doi:10.1016/j.jalz.2011.03.003 21514248 10.1016/j.jalz.2011.03.003 PMC3220946

[30] Heikal SA , Fawi G , Khedr EM , et al. The Egyptian Dementia Network (EDN): baseline characteristics from the first dementia registry in an African Arab country. Alzheimers Dement. 2025;21:e70770. doi:10.1002/alz.70770 41216919 10.1002/alz.70770 PMC12603777

[31] Yang H , Yim D , Park MH . Converting from the Montreal Cognitive Assessment to the Mini‐Mental State Examination‐2. PLoS One. 2021;16:e0254055. doi:10.1371/journal.pone.0254055 34237113 10.1371/journal.pone.0254055 PMC8266092

[32] Aracri F , Bianco MG , Quattrone A , Sarica A . Bridging the gap: missing data imputation methods and their effect on dementia classification performance. Brain Sci. 2025;15:639. doi:10.3390/brainsci15060639 40563810 10.3390/brainsci15060639 PMC12190219

[33] Fjell AM , Rogeberg O , Sørensen Ø , et al. Reevaluating the role of education on cognitive decline and brain aging in longitudinal cohorts across 33 Western countries. Nat Med. 2025;31:2967‐2976. doi:10.1038/s41591‐025‐03828‐y 40721513 10.1038/s41591-025-03828-y PMC12888805

[34] Dubois B , Feldman HH , Jacova C , et al. Research criteria for the diagnosis of Alzheimer's disease: revising the NINCDS‐ADRDA criteria. Lancet Neurol. 2007;6:734‐746. doi:10.1016/S1474‐4422(07)70178‐3 17616482 10.1016/S1474-4422(07)70178-3

[35] Palma LF , Bom RC , Pulgatti KL , et al. Factors influencing the cognitive performance in cognitively unimpaired older adults and people living with Alzheimer's disease: insights from a middle‐income Latin American country. Appl Neuropsychol Adult. 2025:1‐11. doi:10.1080/23279095.2025.2500649 10.1080/23279095.2025.2500649 40338725

[36] Ageing in the ArAb region: StAtiSticAl trendS And Policy PerSPectiveS. 2017.

[37] Ahmad MdA , Kareem O , Khushtar M , et al. Neuroinflammation: a potential risk for dementia. Int J Mol Sci. 2022;23:616. doi:10.3390/ijms23020616 35054805 10.3390/ijms23020616 PMC8775769

[38] Czaja SJ , Moxley JH , Rogers WA . Social support, isolation, loneliness, and health among older adults in the PRISM randomized controlled trial. Front Psychol. 2021;12:4307. doi:10.3389/fpsyg.2021.728658 10.3389/fpsyg.2021.728658 PMC8525598 34675843

[39] Mowafi S , Moustafa SA , Wahdan M , Heikal S , Othman M , Salama M . Dementia in the MENA region uncharted challenges and emerging insights a literature review. NPJ Dement. 2025;1:1‐15. doi:10.1038/s44400‐025‐00009‐z

[40] Silva J , Mesquita TCR , Aires URV , Silva DDD . Spatial variability of water electrical conductivity and its implications for agricultural planning. J Irrig Drain Eng. 2019;145:05019010. doi:10.1061/(ASCE)IR.1943‐4774.0001424

[41] Murman DL . The impact of age on cognition. Semin Hear. 2015;36:111. 27516712 10.1055/s-0035-1555115 PMC4906299

[42] Parrón T , Requena M , Hernández AF , Alarcón R . Association between environmental exposure to pesticides and neurodegenerative diseases. Toxicol Appl Pharmacol. 2011;256:379‐385. 21601587 10.1016/j.taap.2011.05.006

[43] Kulcsárová K , Piel JHA , Schaeffer E . Environmental toxins in neurodegeneration—a narrative review. Neurol Res Pract. 2025;7:93. doi:10.1186/s42466‐025‐00452‐6 41250117 10.1186/s42466-025-00452-6 PMC12625418

[44] Clouston SAP , Hanes DW , Yazdi MD . Accuracy of pattern‐based dementia diagnostic protocols: using longitudinal data to infer etiology of Alzheimer's disease and related dementias compared to stroke or normal aging. Alzheimers Dement. 2025;17:e70211. 10.1002/dad2.70211 PMC12620074 41250667

[45] Bratsberg B , Harris JR , Skirbekk V , et al. Occupational social interaction is associated with reduced dementia risk: the HUNT study. Psychol Aging. 2025;41(1):12‐24. doi:10.1037/pag0000940 41021477 10.1037/pag0000940 PMC12670332

[46] Rajan KB , Weuve J , Barnes LL , McAninch EA , Wilson RS , Evans DA . Population estimate of people with clinical Alzheimer's disease and mild cognitive impairment in the United States (2020‐2060). Alzheimers Dement. 2021;17:1966‐1975. doi:10.1002/alz.12362 34043283 10.1002/alz.12362 PMC9013315

[47] Moriarty F , Savva GM , Grossi CM , et al. Cognitive decline associated with anticholinergics, benzodiazepines and Z‐drugs: findings from The Irish Longitudinal Study on Ageing (TILDA). Br J Clin Pharmacol. 2021;87:2818‐2829. doi:10.1111/bcp.14687 33270264 10.1111/bcp.14687

[48] Ferri CP . Global prevalence of dementia. Alzheimers Dement. 2009;5:118.

[49] Sayed HA , Egypt's Demographic Opportunity Preliminary Assessment based on 2017 Census. UNFPA. 2018.

